# A cross-sectional survey on community pharmacists readiness to fight COVID-19 in a developing country: knowledge, attitude, and practice in Lebanon

**DOI:** 10.1186/s40545-021-00327-6

**Published:** 2021-06-11

**Authors:** Rony M. Zeenny, Ahmad Dimassi, Hala Sacre, Ghada El Khoury, Aline Hajj, Rita Farah, Hind Hajj, Nathalie Lahoud, Marwan Akel, Souheil Hallit, Pascale Salameh

**Affiliations:** 1 Pharmacy Department, American University of Beirut Medical Center, Beirut, Lebanon; 2 School of Pharmacy, Lebanese International University, Beirut, Lebanon; 3 INSPECT-LB: Institut National de Santé Publique, d’Épidémiologie Clinique et de Toxicologie, Beirut, Lebanon; 4 School of Pharmacy, Lebanese American University, Byblos, Lebanon; 5 Faculty of Pharmacy, Saint-Joseph University, Beirut, Lebanon; 6 Laboratory of Pharmacology, Clinical Pharmacy and Quality Control of Drugs, Pôle Technologie-Santé (PTS), Faculty of Pharmacy, Saint-Joseph University, Beirut, 1107 2180 Lebanon; 7 Faculty of Pharmacy, Lebanese University, Hadat, Lebanon; 8 CERIPH: Center for Research in Public Health, Pharmacoepidemiology Surveillance Unit, Faculty of Public Health, Lebanese University, Fanar, Lebanon; 9 Faculty of Medicine and Medical Sciences, Holy Spirit University of Kaslik (USEK), Jounieh, Lebanon; 10 University of Nicosia Medical School, Nicosia, Cyprus

**Keywords:** COVID-19, Knowledge, Attitude, Practice, Protective measures, Patient service, Community pharmacy

## Abstract

**Background:**

Lebanon, a developing Middle Eastern country, was hit by the COVID-19 pandemic that occurred amid a severe national economic crisis. Community pharmacists are responsible for dispensing appropriate medications and products in addition to counseling, informing and educating the public, and promoting disease prevention and infection control.

**Objective:**

This study aimed to assess community pharmacists' knowledge, attitude, and practice towards the COVID-19 pandemic and evaluate behavior changes and safety measures.

**Methods:**

An anonymous and standardized online questionnaire in English was disseminated via social media platforms to Lebanese community pharmacists. The questionnaire consisted of 95 items designed as Likert-scales and multiple-choice questions divided into four different sections: socio-demographic characteristics, knowledge-based, attitude-based, and pharmacy practice questions. Descriptive statistical analysis was used to summarize the demographic characteristics, and indices were created for knowledge, attitude, and practice by computing the correct answers for each section.

**Results:**

A total of 310 questionnaires were completed. Around 61% have expressed their fear of getting infected with COVID-19 due to occupational exposure. The respondents were able to answer 80–90% of the knowledge-based questions of the survey. A more careful/anxious attitude, but not knowledge, was associated with overall better practice (*p* = 0.03). Also, respondents stated that they are dispensing protective equipment items such as masks (87%), gloves (60%), and sanitizers (77%) in small quantities due to limited availability.

**Conclusion:**

Our findings revealed an adequate level of knowledge and good practice towards COVID-19 among Lebanese community pharmacists. Their fears of contracting the virus and compromising the safety of those around them are justified. However, their supply of protective equipment is limited.

**Supplementary Information:**

The online version contains supplementary material available at 10.1186/s40545-021-00327-6.

## Background

On December 31, 2019, the world witnessed the emergence of a novel human coronavirus, SARS-CoV-2 [1]. Its associated disease, the 2019 Coronavirus Disease (COVID-19), was declared a pandemic by the World Health Organization (WHO) on March 11 [2]. In April 2020, the overall case-fatality rate was around 3.4% [3], but reached 8.0% in patients aged 70 to 79 and 14.8% in those over 80 [4]. The COVID-19 typically spreads from person-to-person by respiratory droplets produced while coughing or sneezing; transmission also occurs through contaminated surfaces. The global impact of COVID-19 has been profound, and the public health threat it represents is the most serious seen in a respiratory virus since the 1918–1919 H1N1 influenza pandemic [5]. Sequentially, several global interventions were proposed and implemented to flatten the spread curve and avoid overwhelming healthcare facilities. Travel restrictions, closing schools, colleges, bars, and restaurants, banning crowded events, and social distancing were all part of the global lockdown strategy [6].

The burden of COVID-19 is a challenge for the health authorities, who had to respond swiftly to avoid the healthcare system being overwhelmed [7]. Healthcare professionals across all settings are frontline warriors caring for and communicating with patients in a context of high uncertainty, with an increased risk of infection coupled with physical and mental exhaustion. In affected countries, community pharmacists are essential healthcare providers and often the first contact point for health information. They also play a significant role in dispensing medicines and counseling patients with acute and chronic illnesses [8, 9]. Death due to COVID-19 has hit pharmacists from Spain, China, and Italy while serving their community [8]. Therefore, community pharmacists, like all other frontlines healthcare providers, need to be equipped with knowledge and tools to protect themselves and communicate effectively with patients about ways to limit the spread of COVID-19.

Several national and international professional organizations [8, 10–12] have made available supporting resources to provide pharmacists with information, assisting them in safely operating their pharmacy and meeting community needs during the COVID-19 pandemic. Recommendations offered to pharmacists can be summarized as follows: (1) reducing the number of patients in pharmacy at any one time, establishing a safe distance of at least 1.5 m between people by placing marks on the ground, and adding a plastic or Perspex shield in front of the dispensing area and counters; (2) limiting access to parts of pharmacy and restricting patient movement, including prohibiting patients to self-select products; (3) limiting entry to the pharmacy and referring patients with respiratory symptoms to the COVID-19 national hotline for triage and advice; (4) providing hand sanitizer on counters for visitors and securing easy access to soap and water and hand sanitizers to staff; (5) cleaning and disinfecting frequently touched surfaces [13].

Lebanon is a developing Middle Eastern country with a challenging economic and financial situation that had started well before the COVID-19 pandemic. The first case of COVID-19 in Lebanon was identified on February 21, and 632 cases and 20 deaths were reported by April 13. Early governmental efforts, applied by the Ministry of Public Health (MOPH), have focused on disease containment and curve flattening [14]. In-hospital healthcare professionals were required to wear appropriate personal protective equipment, but nothing was mentioned for those working outside hospitals. Along the same lines, community pharmacists (4000 distributed across Lebanon) are heavily impacted by the COVID-19 pandemic, working in less than optimal conditions with high-risk pharmacy staff exposure [10]. In some overcrowded regions, they are assisted by fifth- and sixth-year pharmacy students who start working before graduation to gain experience.

The responsibilities of community pharmacists are numerous and include dispensing appropriate medications and products, storing and supplying adequate stocks of medicines, devices, masks, in addition to counseling, informing, and educating the public, triaging and referring patients, and promoting disease prevention and infection control [15, 16]. The national association of pharmacists known as the Order of Pharmacists of Lebanon (OPL) had issued a brief communiqué, asking the community pharmacists to add a shield on the counter between them and the patient or to deliver medications through a window opened in the pharmacy door (like in drive-through), and to restrict the entry of sick patients or persons with a history of travel or contact with a known COVID-19 case within the past 10 days [10]. Our team also adapted and prepared documents to be diffused to community pharmacists to help them with their critical endeavors (documents available at https://inspect-lb.org/covid-19-information).

This study aimed to assess community pharmacists' knowledge, attitude, and practice towards the COVID-19 pandemic and evaluate behavior changes and safety measures.

## Methods

### Study design and procedure

A descriptive, cross-sectional survey was carried out from March 18 through April 3, 2020, among licensed pharmacists and fifth- and sixth-year pharmacy students working in community pharmacies across all governorates of Lebanon (Beirut, Mount Lebanon, North, South, and Beqaa). The authors assumed that senior pharmacy students working in community pharmacies might be more technology savvy than senior pharmacists when filling online surveys. Data collection from eligible participants started 2 days after the sanitary lockdown (March 16). A snowball sampling method was applied using a self-reported online pretested questionnaire created on Google forms [17] because of the inability to reach pharmacists due to the lockdown imposed by the Lebanese government and the incapacity to request the e-mail database from the OPL since it was closed.

The questionnaire was tested on ten community pharmacists before the link was sent to pharmacists and senior pharmacy students through social media platforms (WhatsApp groups, Facebook, Twitter, etc.); it required approximately 12 min to complete.

Licensed pharmacists practicing in settings other than the community and those not currently working in Lebanon were not included in the survey.

### Ethical approval

The Ethics and Research Committee at the Psychiatric Hospital of the Cross approved the study (HPC-010-2020). The online voluntary and anonymous questionnaire secured the confidentiality of the participants. The survey did not collect any information exposing the privacy of the participants.

### Sample size

The minimum sample size was calculated using Epi Info, based on the presence of 4000 community pharmacists in Lebanon, a confidence level of 95%, a precision level of 5%, and assuming that 75% of the community pharmacists are familiar with the recommendations of the OPL [10] diffused to all pharmacists and those of the International Pharmaceutical Federation (FIP) [8] available online. Accordingly, the minimum required sample for this survey was 269.

### Questionnaire design

A group of ten pharmacists with a long experience in community pharmacy and academia developed the questionnaire based on a thorough review of the available and related literature on the topic, in addition to the latest recommendations from professional organizations. The English questionnaire was piloted and reviewed in a group of ten community pharmacists, randomly chosen, to help understand and clarify questions, thus producing reliable results. It is noteworthy that the vast majority of pharmacists speak English, which is the language adopted by the OPL for their continuing education. Sequentially, the review generated minor changes; thus, the validation database was reset before the link was disseminated to pharmacists.

The questionnaire consisted of 95 items designed as Likert-scales and multiple-choice questions divided into four different sections: socio-demographic characteristics (1–10, including gender, age, location, etc.), knowledge questions (11–28), attitude questions (29–35), pharmacy practice (safety of pharmacy staff: 36–68 and service to patients: 69–95). At the end of the questionnaire, a comment box was added for suggestions and comments. In the knowledge section, multiple answers were possible for some questions. The full questionnaire is available in the appendix (Additional file 1: Appendix A). Knowledge, attitude, and practice scores were computed by summing the correct answers for each section.

### Data management and statistical analysis

Data were checked for completeness and consistency, and the analysis was performed using IBM SPSS Statistics for Windows version 23.0 (IBM Corp., Armonk, NY, USA). Descriptive statistical analysis was used to summarize the demographic characteristics, and indices were created for knowledge, attitude, and practice by computing the correct answers of each section (a response was considered exact when it corresponded to the information available in the literature at the time of data collection). Continuous data were expressed as means and standard deviations (M ± SD), and the Student’s t-test, ANOVA test, or Pearson correlation were used for comparisons. Categorical data were reported as absolute numbers and proportions. A value of *p* < 0.05 was considered significant in all cases.

## Results

### General characteristics of community pharmacists and pharmacies

A total of 310 community pharmacists participated in the survey (mean age: 31.85 years ± SD 7.99, 69% females). The highest percentage of respondents was from Mount Lebanon. Of the total sample, 53% were employee pharmacists (including 3(1%) senior pharmacy students), with 73% of respondents working on a full-time basis. As for the pharmacy characteristics, the mean surface area was 108 square meters, with 54% receiving 10–50 patients asking questions about COVID-19 and 42% having two persons together on duty, in general. A significantly higher mean practice score was found in pharmacy owners compared to employees (37.59 vs. 35.77; *p* = 0.026), while a higher mean attitude score was found in those working part-time compared to full time (10.17 vs. 8.85; *p* = 0.007) (Table 1).

**Table 1:** Characteristics of Lebanese community pharmacist and pharmacies

Characteristics of pharmacist and pharmacy	Total = 310	Knowledge	Attitude	Practice
Age (mean ± SD)	31.85 ± 7.99	*r* = − 0.06; *p* = 0.308	*r* = − 0.07; *p* = 0.222	*r* = − 0.044; *p* = 0.437
Gender				
Males	95 (30.6%)	25.36 ± 3.90	8.78 ± 3.67	36.65 ± 6.52
Females	215 (69.4%)	26.06 ± 3.62	9.39 ± 3.92	36.84 ± 7.44
*p*		0.124	0.195	0.835
Muhafazat				
Beirut	72 (23.2%)	25.55 ± 3.31	9.48 ± 3.87	35.90 ± 6.76
Mount Lebanon	150 (48.4%)	26.08 ± 3.74	9.35 ± 3.69	36.78 ± 7.10
North	26 (8.4%)	26.11 ± 4.02	9.92 ± 4.02	36.88 ± 7.63
South	45 (14.5%)	25.49 ± 3.77	8.22 ± 4.13	38.15 ± 6.92
Bekaa	17 (5.5%)	25.53 ± 4.65	8.29 ± 4.04	36.70 ± 9.34
*p*		0.817	0.253	0.604
Years of experience in community pharmacy	7.51 ± 8.49	*r* = − 0.04; *p* = 0.470	*r* = − 0.04; *p* = 0.461	*r* = − 0.06; *p* = 0.254
Owner of the Community Pharmacy	145 (46.8%)	25.68 ± 3.78	8.96 ± 3.74	37.59 ± 6.73
Employee at the Community Pharmacy	165 (53.2%)	25.89 ± 3.64	9.35 ± 3.93	35.77 ± 7.44
*p*		0.629	0.371	**0.026**
Working on a Part-time basis	84 (27.1%)	25.47 ± 3.82	10.17 ± 3.90	36.82 ± 8.47
Working on a Full-time basis	226 (72.9%)	25.98 ± 3.67	8.85 ± 3.78	36.76 ± 6.63
*p*		0.285	**0.007**	0.951
Per day, how many patients come into the pharmacy asking you about the COVID-19 virus?				
< 10	36 (11.6%)	26.14 ± 3.34	9.11 ± 4.14	34.36 ± 6.49
10–50	168 (54.2%)	25.99 ± 3.78	9.14 ± 4.01	37.27 ± 6.44
51–100	62 (20.0%)	25.16 ± 3.88	9.84 ± 3.44	37.37 ± 8.48
> 100	44 (14.2%)	26.03 ± 3.52	8.64 ± 3.56	36.04 ± 8.03
*p*		0.444	0.442	0.122
How many pharmacists/technicians are on duty at any single point in time at the pharmacy?				
1	118 (38.1%)	25.80 ± 3.85	8.79 ± 3.86	36.42 ± 6.58
2	130 (41.9%)	25.82 ± 3.68	9.44 ± 3.80	37.34 ± 7.18
3	41 (13.2%)	25.51 ± 3.47	9.05 ± 4.29	37.05 ± 7.92
> 3	21 (6.8%)	26.95 ± 3.69	10.38 ± 3.07	34.81 ± 8.66
*p*		0.530	0.272	0.437

Numbers in bold indicate significant *p*-values; numbers are presented as frequency (percentage) or mean ± standard deviation

### Knowledge related to COVID-19

The respondents could answer 80–90% of the knowledge-based questions regarding preventive measures, duration of quarantine, testing-related matters, the alcohol concentration to kill viruses, acetaminophen use, and the MOPH hotline number. However, 10% to 20% were not able to answer the knowledge-based questions, while a few pharmacists declared not knowing the answers to the questions asked (< 1%) (Table 2). Also, pharmacists were less familiar with other more sensitive matters (50–70%), such as the risk of attending funerals of a person who died from COVID-19, the number of PCR tests necessary to release a person from quarantine, contact with pets, and currently used treatments (all options were considered acceptable except steroids and NSAIDS since their use is still controversial) (Table 2).

**Table 2:** Knowledge of Lebanese community pharmacists related to COVID-19

Questions	*N* = 310 (%)
Which of the following is true about COVID-19? 87^a^	
**Person-to-person transmission can occur by droplets**	302 (97.4%)
**Transmission can be airborne**	85 (27.4%)
Most common symptoms include fever, diarrhea, and dyspnea	175 (56.5%)
I do not know	2 (0.6%)
Effective methods to protect yourself from potentially infected patients?^a^	
**Wearing a surgical mask during the shift**	263 (84.8%)
**Washing hands frequently with soap and water**	306 (98.7%)
**Rubbing hands with alcohol-based gel**	285 (91.9%)
**Wearing gloves and changing them frequently**	237 (76.5%)
**Maintain a physical separation of at least 1.5 m**	300 (96.8%)
**Limit the time interacting with an individual who has respiratory symptoms**	260 (83.9%)
I do not know	1 (0.3%)
For how long should a person be isolated in case of COVID-19 infection suspicion (mild symptoms or contact with an infected person)?^a^	
7 days	1 (0.3%)
10 days	0
**14 days**	287 (92.6%)
**20 days**	16 (5.2%)
** > 20 days**	4 (1.3%)
I do not know	2 (0.6%)
Can someone who was quarantined for COVID-19 spread the illness to others?	
No, if the quarantine period is less than 14 days	15 (4.8%)
**No, if the quarantine period is 14 days or more**	275 (88.7%)
I do not know	20 (6.5%)
What are the steps to take to protect yourself?^a^	
Wash your hands with soap and water for at least 10 s	19 (6.1%)
**Wash your hands with soap and water for at least 20 s**	304 (98.1%)
**Avoid close contact; put a distance from other people (1.5–2 m)**	299 (96.5%)
**Wear a facemask and stay home if you have any respiratory symptom**	
No need to clean and disinfect solid objects (tables, doorknobs, desks, phones)	275 (88.7%)
**Can a person test negative and later test positive for COVID-19?**	9 (2.9%)
No	8 (2.6%)
**Yes**	290 (93.5%)
I do not know	12 (3.9%)
If a suspected person tests negative but has no symptoms:	
It is definitely a true negative	39 (12.6%)
**It can be a false negative in the pre-symptomatic phase**	248 (80%)
I do not know how to interpret this test result, I refer to a specialist	39 (12.6%)
Is the person at risk if he/she goes to the funeral of someone who died of COVID-19?	
**Yes, since he will meet the dead person close contacts**	208 (67.1%)
No known risk currently	69 (22.3%)
I do not know	33 (10.6%)
When can confirmed COVID-19 cases be released from quarantine?^a^	
Following one negative PCR test after the resolution of symptoms	29 (9.4%)
**Following two negative PCR tests 24 h apart after the resolution of symptoms**	192 (61.9%)
Following four negative PCRs on 3 consecutive days after symptoms resolution	47 (15.2%)
I do not know	42 (13.5%)
Should I avoid contact with pets or other animals?	
**No**	129 (41.6%)
Yes	145 (48.8%)
I do not know	36 (11.6%)
Which of the below products are you recommending to patients to disinfect?	
**Alcohol 60%**	44 (14.2%)
**Alcohol 70%**	297 (95.8%)
Alcohol 95%	52 (16.8%)
What are the most common symptoms related to COVID-19?	
Fever, productive cough, rhinorrhea	6 (1.9%)
**Fever, dry cough, dyspnea**	294 (94.8%)
Fever, diarrhea, pharyngitis	9 (2.9%)
None of the above	0
I do not know	1 (0.3%)
Indicate which of these options can be used to treat COVID-19 to date?^a^	
**Acetaminophen**	272 (87.7%)
Non-steroidal anti-inflammatory drugs (NSAIDs)	13 (4.2%)
Corticosteroids	18 (5.8%)
**Symptomatic respiratory relief (inhalers)**	140 (45.2%)
**Lopinavir/ritonavir (initially for HIV)**	104 (33.5%)
**Chloroquine/remdesivir in combination**	134 (43.2%)
**Tocilizumab (initially for rheumatoid arthritis)**	52 (16.8%)
Intravenous high-dose vitamin C is recommended for COVID-19 treatment?	
**True**	47 (15.2%)
False	155 (50.0%)
I don’t know	108 (34.8%)
What is the MOPH hotline number?	
112	14 (4.5%)
125	11 (3.5%)
**1214**	277 (89.4%)
1515	8 (2.6%)

Correct answers in bold. ^a^More than one answer can be correct. Numbers are presented as frequency (percentage)

### Information about COVID-19

The majority of pharmacists (88.7%) reported getting information about COVID-19, mainly for 1–2 h per day (46%), some for less than one hour (35%), and some not at all (3.9%); as for sources of information, the most trusted were the WHO (87%), MOPH (59%), and CDC (48%). However, around 46% still relied on unspecified websites, 34% on television, 17.4% on Facebook, and 5.5% on family and friends (Table 3).

**Table 3:** Information about COVID-19

Questions	*N* = 310(%)
Do you have time to get information regarding the COVID-19 outbreak?	
3–4 h/day	47 (15.2%)
1–2 h/day	142 (45.8%)
< 1 h/day	109 (35.2%)
Not at all	12 (3.9%)
Where do you get your information on COVID-19 from?	
CDC website	149 (48.1%)
Ministry of Public Health website (MOPH)	183 (59.0%)
World Health Organization (WHO)	268 (86.5%)
Infectious Disease Society of America (IDSA)	13 (4.2%)
Media website/Internet	142 (45.8%)
Facebook	54 (17.4%)
Friends/family members	17 (5.5%)
Television	105 (33.9%)
Other, specify: Twitter, NEJM, JAMA, Medscape	4 (1.3%)

Numbers are presented as frequency (percentage)

### Additional matters related to MOPH Hotline and other comments

Very few pharmacists tried to reach the MOPH COVID-19 call center (11.3%), and 36.8% reported having attended an awareness session on COVID-19. In the last open-ended question, some pharmacists expressed many needs, including information and clear recommendations, brochures to give for patients/customers, training on how to protect from COVID-19, in addition to information on how to fight fake news and panic and how to improve mental health.

### The attitude of the Lebanese community pharmacists towards COVID-19

Most respondents (82%) were concerned about getting infected and their families due to their professional exposure. Almost half of them declared being exhausted because of the pandemic but claimed that their stress did not affect their professional duties nor their family and staff relationships, and very few employee pharmacists (13%) expressed their desire to quit their jobs. However, only 41.6% of pharmacies have implemented icebreaking or energizing actions to mitigate staff stress (Table 4).

**Table 4:** The attitude of the Lebanese community pharmacists towards COVID-19

Questions	Never	Rarely	Often	Always
Are you afraid of getting infected with COVID-19 due to occupational exposure?	30 (9.7%)	92 (29.7%)	120 (38.7%)	68 (21.9%)
Are you afraid your family members get infected because of your occupational exposure?	7 (2.3%)	49 (15.8%)	94 (30.3%)	160 (51.6%)
Do you feel depressed/exhausted due to the current pandemic?	42 (13.5%)	99 (31.9%)	123 (39.7%)	46 (14.8%)
Are stress feelings affecting your duties (counseling, education, assessment)?	138 (44.5%)	110 (35.5%)	41 (13.2%)	21 (6.8%)
Are stress feelings affecting your relationship with your staff and family members?	113 (36.5%)	116 (37.4%)	61 (19.7%)	20 (6.5%)
Does any of your staff declare wanting to leave work due to COVID-19 fear?	211 (68.1%)	60 (19.4%)	27 (8.7%)	12 (3.9%)
Do you implement specific icebreaking or energizing actions in your pharmacy to mitigate your staff stress?	63 (20.3%)	118 (38.1%)	90 (29.0%)	39 (12.6%)

Numbers are presented as frequency (percentage)

### The practice of community pharmacists regarding safety

While two-thirds of community pharmacists reported following appropriate safety recommendations, one-third still does not apply many protective measures, such as restricting the number of persons entering the pharmacy, installing a protective shield or a mark-up barrier, wearing a mask or gloves during work shifts. Also, more than 80% do not use a basket to collect money or credit cards from the customer.

As for shifts management, the vast majority (more than 75%) are still operating as usual, with no reduction in timing and no change or rotation when shifts overlap. Nevertheless, none was tested positive for COVID-19 till now. Those who wore masks would change it after a mean of 4.5 h; for gloves, it would be after 2 h. Although more than 70% declared being able to frequently wash or rub their hands, avoid contact with customers, and touching their face, a lower percentage could keep a safe distance from their work colleagues; also, about 35% do not stay home even if they are not feeling well (Table 5).

**Table 5:** The practice of community pharmacists regarding safety

Questions	No		Yes	
Was any of your staff members tested positive for COVID-19?	310 (100%)		0	
Did you put a sign outside to restrict patients inside the pharmacy to one only at a time?	103 (33.2%)		207 (66.8%)	
Did you put a sign directing the patient not to enter the pharmacy in case of symptoms or exposure?	71 (22.9%)		239 (77.1%)	
If yes, is this sign being read by patients?	62 (25.9%)		177 (74.1%)	
Did you make hand gel (hydro-alcoholic) available for patients to use before approaching the counter?	52 (16.8%)		258 (83.2%)	
Did you install a Plexiglas/Glass protective shield?	73 (23.5%)		237 (76.5%)	
Do you restrict the number of patients’ entering the pharmacy	95 (30.6%)		215 (69.4%)	
Do you deliver medications through a window while forbidding all patients/clients from entering?	193 (62.3%)		117 (37.7%)	
Did you create any mark-up barrier for patients to stay away at least 1.5 m from you?	114 (36.8%)		196 (63.2%)	
Are you wearing a mask while performing your job at the pharmacy	58 (18.7%)		252 (81.3%)	
Are you required to wear a mask while performing your job at the pharmacy	86 (27.7%)		224 (72.3%)	
Are you wearing gloves while performing your job at the pharmacy	112 (36.1%)		198 (63.9%)	
Are you required to wear gloves while performing your job at the pharmacy	145 (46.8%)		165 (53.2%)	
Are you wearing goggles/glasses to protect your eyes while performing your job at the pharmacy	249 (80.3%)		61 (19.7%)	
Are you still working as a full team as before COVID-19?	69 (22.3%)		241 (77.7%)	
If you are alternating schedule, were you asked to use vacation days?	248 (80.0%)		62 (20.0%)	
Did you organize yourself in a way to serve your patients outside the community pharmacy? (delivery for example)	229 (73.9%)		81 (26.1%)	
Do you prefer patients to pay by credit/debit card?	196 (63.2%)		114 (36.8%)	
Are you using a basket to collect money from patients to avoid direct contact?	263 (84.8%)		47 (15.2%)	
Did the working hours decrease for staff to decrease exposure?	225 (72.6%)		85 (27.4%)	
Did the working hours decrease for pharmacy owners to decrease exposure?	231 (74.5%)		79 (25.5%)	
Were rotations changed in a way to decrease exposure?	221 (71.3%)		89 (28.7%)	
For how long do you put your face mask before changing it (hours)?	4.44 (3.74)			
For how long do you put your gloves before changing them (hours)?	2.14 (2.22)			

Additional questions	Never	Rarely	Often	Always
Are you able to wash your hands during your shift?	7 (2.3%)	20 (6.5%)	97 (31.3%)	186 (60.0%)
Are you able to rub your hands with hydro-alcoholic gel during your work shift?	2 (0.6%)	5 (1.6%)	73 (23.5%)	230 (74.2%)
Are you able to maintain social distancing of at least 1.5 m from patients	3 (1.0%)	21 (6.8%)	127 (41.0%)	159 (51.3%)
Are you able to maintain social distancing of at least 1.5 m from work colleagues	39 (12.6%)	99 (31.9%)	100 (32.3%)	72 (23.2%)
Are you able to avoid touching your eyes, nose, and mouth?	11 (3.5%)	52 (16.8%)	137 (44.2%)	110 (35.5%)
Are you able to avoid close contact with people/patients with respiratory symptoms?	9 (2.9%)	22 (7.1%)	110 (35.5%)	169 (54.5%)
Are you able to stay home if not feeling well?	48 (15.5%)	58 (18.7%)	88 (28.4%)	116 (37.4%)

### Provision of protective devices and supplements

The majority agreed that they had masks, gloves, and hand sanitizers to sell, but only in limited quantities, and did not offer masks free of charge. Almost all pharmacists are facing supply delays, rising prices, and pressure from suppliers to pay in cash and after short periods (Table 6).

**Table 6:** Protective devices supply

Questions about supply	No	Yes
Do you still have enough masks to sell to patients?	121 (39.0%)	189 (61.0%)
If yes, are you selling them one by one/in small quantities?	41 (13.2%)	269 (86.8%)
Do you still have enough gloves to sell to patients?	81 (26.1%)	229 (73.9%)
If yes, are you selling them one by one/ in small quantities?	124 (40.0%)	186 (60.0%)
Do you still have enough sanitizers to sell?	36 (11.6%)	274 (88.4%)
If yes, are you selling them one by one/ in small quantities?	73 (23.5%)	237 (76.5%)
Do you offer masks free of charge to patients?	231 (74.5%)	79 (25.5%)
Are you facing a delay in the supply of masks, gloves, or hand gels from suppliers?	39 (12.5%)	271 (87.4%)
Are you facing an increase in the price of the masks, gloves, and hand gels from the supplier in a regular manner?	7 (2.3%)	303 (97.7%)
Are you facing pressure from suppliers to pay cash or in a short period?	27 (8.7%)	283 (91.3%)

Numbers are presented as frequency (percentage)

As for the supplements, pharmacists are dispensing them when patients ask to boost their immune system against COVID-19. The most sought are vitamin C (more than 90%), zinc-containing supplements (70%), multivitamins (50%), vitamin D (less than 20%), and herbal supplements (< 5%).

### Practice related to patients’ advice

When patients come to the pharmacy with respiratory symptoms, almost all pharmacists ask questions related to their travel history and contact with a confirmed case or with travelers from an affected area; however, 72% do not offer them any awareness brochure (Table 7).

**Table 7:** Questions about patients’ advice

If a patient comes to your pharmacy with respiratory symptoms	Never	Rarely	Often	Always
Do you ask questions about travel history?	9 (2.9%)	21 (6.8%)	92 (29.7%)	188 (60.0%)
Do you ask the patient if he had contact with a confirmed case COVID-19	17 (5.5%)	22 (7.1%)	76 (24.5%)	195 (62.9%)
Do you ask him if he had contact with travelers coming from the affected area	13 (4.2%)	26 (8.4%)	84 (27.1%)	187 (60.3%)
Do you offer patients awareness brochures?	No: 223 (71.9%)		Yes: 87 (28.1%)	

How do you behave in these situations?	Call the physician	Call the MOPH	Laboratory for testing	Preventive advice^a^
Flu-like symptoms with, during the last 14 days, history of travel to infected areas/Or contact with a COVID-19 case	80 (25.8%)	196 (63.2%)	140 (45.2%)	17 (37.7%)
Flu-like symptoms without a history of travel to infected areas or contact with confirmed COVID-19 case	165 (53.2%)	51 (16.5%)	79 (25.5%)	162 (52.3%)
Corona suggestive symptoms (fever, dry cough, dyspnea)	147 (47.4%)	196 (63.2%)	158 (51.0%)	127 (41.0%)
Patient with a Positive COVID-19 PCR test, with or without respiratory symptoms	110 (35.5%)	266 (85.8%)	59 (19.0%)	120 (38.7%)
Patient panicking due to suspected exposure	106 (34.2%)	102 (32.9%)	159 (51.3%)	184 (59.4%)
Patient panicking without symptoms or exposure	62 (20.0%)	7 (2.3%)	55 (17.7%)	264 (85.2%)

^a^Preventive advice included social distancing, hygiene measures, and quarantine if necessary; numbers are presented as frequency (percentage)

The most frequent behaviors reported by pharmacists in case of flu-like symptoms with a positive history suggestive of COVID-19 was to call the MOPH (63%), followed by referring the patient to a laboratory for testing (45%), giving preventive advice (40%), and calling the physician (26%); close percentages were reported when patients had symptoms suggestive of COVID-19. In case of flu-like symptoms with no history suggestive of COVID-19, half of pharmacists would call the physician and/or give preventive advice, 25% would still refer for laboratory testing, and 17% would call the MOPH (Table 7).

If a patient has a positive PCR test, 86% of pharmacists would call the MOPH, with about one-third calling the physician or giving advice, and 19% still referring for laboratory testing. In case a customer is panicking because of suspected exposure, 50 to 60% of pharmacists would give him preventive advice or refer him to a laboratory for testing, while around 30% would call the physician or the MOPH. In case a customer is panicking without any symptoms or suggestive history, 85% would give preventive advice, and 20% would still refer to a physician or a laboratory for testing (Table 7). It is noteworthy that respondents did not leave any comments or suggestions.

### Bivariate analysis of factors associated with knowledge, attitude, and practice

The respective means and standard deviation of indices were as follows: 25.85 (3.72) for knowledge (max possible 42), 9.21 (3.86) for careful attitude (max 21), 21.41 (4.38) for protective practice (max 28), and 22.27 (8.61) for patient service-related practice (max 30). Neither the socio-demographic variables nor the attitude and practice indices were correlated with the knowledge index. A significantly higher mean attitude index was found among part-time pharmacists compared to full-timers (10.17 vs. 8.85; *p* = 0.007), and a significantly higher overall mean practice index was found among employers compared to employees (37.59 vs. 35.76; *p* = 0.026). Furthermore, a more careful/anxious attitude was weakly but significantly associated with better overall practice (*r* = 0.120; *p* = 0.03).

## Discussion

To the best of our knowledge, this is the first rapid appraisal study in Lebanon, examining the knowledge, attitude, and practice towards COVID-19 among Lebanese community pharmacists [18]. To date, the COVID-19 outbreak is considered an emerging disease, and pharmacists are at high risk of contracting the infection when attending their shifts at the community pharmacy setting, yet few studies examined the knowledge and attitude of health workers towards COVID-19 worldwide.

### Knowledge about COVID-19

This study revealed an adequate overall knowledge among Lebanese community pharmacists. A high percentage of up to 90% was found on the basic knowledge questions related to preventive measures, tests, and quarantine duration, indicating that most respondents are knowledgeable about COVID-19 general information. This finding was expected since this survey was conducted in mid-March, around one month following the announcement of the first COVID-19 case in Lebanon [18] and after several authorities diffused information regarding COVID-19, communicated to the general population through mass media. These results can further be explained by the sample characteristics that included young pharmacists, fresh graduates, and senior pharmacy students, who have better access to social media platforms than their elderly counterparts. Our results are similar to those of Zhong et al. who found an overall knowledge rate of 90% among Chinese residents [19]. Similarly, Huynh et al. showed that healthcare workers, particularly pharmacists, had a high level of knowledge about the COVID-19 outbreak in Vietnam [20].

However, a lower knowledge (up to 70%) was found for more delicate and less addressed questions in the media: a significant percentage of pharmacists do not have the correct information related to the risk of attending funerals, pets contact, number of PCR tests before releasing a person from quarantine, and currently available treatments; this could be attributed to the short period allocated for reading about COVID-19 updates (< 1 h up to 2 h per day) and the limited availability of information about these subjects at the time of data collection and may imply that pharmacists need to allocate more time reading and learning new information about this pandemic.

Consequently, information about sensitive and less addressed points should be more communicated to pharmacists since all of them reported receiving a high number of patients asking for information and advice. In Lebanon, funerals are a sensitive issue for communities and difficult to change from a cultural perspective since it is considered part of the social duties that may override the risk of infection. Advice about pets should also be clear since some people in Lebanon are needlessly abandoning their pets [21] or do not disinfect them properly before they enter the home. Moreover, the respondents did not know about the usefulness of vitamin C supplementation [22] or the relative contraindication of NSAIDs and steroids to treat COVID-19, despite clear recommendations by health authorities to avoid them until the controversy over their use is solved [23]. The lack of appropriate information may be explained by a resistance to continuing education by pharmacists in Lebanon [24]; a more specific example shows that Lebanese community pharmacists had insufficient information about a relatively common disease [25]. Thus, additional efforts would be required in times of crisis.

Our results revealed that pharmacists sought information on COVID-19 from various resources, mainly from the WHO, MOPH, and CDC, and to a lesser extent, from social media, due to the severity of the epidemic and the devastating reports on this public health emergency. Thus, most participants relied on credible resources to raise awareness in the community; however, a significant number still relied on television, Facebook, and even family and friends to get information. In the same line, Huynh et al. found that healthcare workers are more interested in social media to gather knowledge on an emerging infectious disease like COVID-19 than the official website of the Ministry of Health [20]. Moreover, the Lebanese community pharmacists requested the authorities to provide them with additional information, training, and clear recommendations on COVID-19 protection measures outside the hospital setting. This issue should be a primary concern for the Lebanese government and relevant authorities to consider and provide several choices of learning materials and platforms, aiming to update the data on this epidemic and communicate information to pharmacists who lack knowledge or are not current on issues relating to COVID-19. Well-informed pharmacists are expected to protect themselves better and provide more appropriate advice to their patients. It is suggested that the adapted documents prepared by our team be diffused to all Lebanese pharmacists (Available at www.inspect-lb.org/covid-19-information). Finally, our results revealed no significant relationship between overall knowledge and practices, explained by the somewhat homogeneous knowledge and practice of pharmacists, the modest sample size, or information bias (practices affected by social desirability).

### Attitudes towards COVID-19

Although no cases of COVID-19 resulting from contacts with positive patients were reported among community pharmacists in Lebanon, respondents are exhibiting an anxious attitude and careful behavior, both associated with better practice. However, in the long term, this might be risky. Indeed, the majority of pharmacists had justified attitudes of exhaustion and fear of getting the virus or passing it on to family members, consistent with the findings of studies conducted in Vietnam and Taiwan [20, 26]. It is paramount to implement occupational safety measures and reinforce the trust of healthcare workers in the system. These measures would mitigate the potential decline of pharmacists’ availability and productivity (due to anxiety and exhaustion) and the possible increase in infections [26].

### The practice of the Lebanese community pharmacists

The identified practices of community pharmacists in our study provide overall assurance regarding self-protection, disinfection techniques, and avoidance of space overcrowding. A poor practice worth noting is coming to work despite not feeling well, as reported by a group of pharmacists; also, pharmacy owners seemed to adopt better practices than employees. As for patients’ services, appropriate practices were noted, with pharmacists reluctant to decide even for patients panicking without any other suggestive history and referring to physicians and MOPH when unnecessary. Moreover, pharmacists referred to laboratory testing without compelling indication, knowing that Lebanon has a shortage of testing kits; therefore, misinterpretation of results might be deleterious for patients’ health.

Furthermore, pharmacists reported a limitation of protective equipment provision, which could jeopardize both patients’ and pharmacists’ health. Public health authorities should remain vigilant regarding the physical and mental health of the healthcare workers and workplace conditions. Given the underlying economic difficulties and the delays in protective equipment supplies, under-resourced and overworked pharmacists might face the real possibility of catching the infection in a rapidly developing pandemic: the result can be detrimental to the community and the nation. Appropriate knowledge, attitudes, and practices towards coronavirus containment and better protection will ease concerns both for pharmacists and the patients they serve while contributing to breaking the cycle of virus spread in the community. Therefore, public health authorities are urged to maintain implementing and monitoring the containment of COVID-19 and promote measures to protect healthcare workers in general, pharmacists and pharmacy workers in particular, as recommended by the FIP [27].

## Limitations and strengths

This study had some limitations in the interpretation of the knowledge results about COVID-19, which is a new disease, especially since the information changes quickly, and research is frequently updated to understand its different aspects. Given the new and evolving knowledge of the subject matter, some of the valid responses provided in the study are no longer correct/accurate. Additionally, during that time, new information from various health entities was released daily, which could have impacted the answers and the accuracy of the findings/conclusions of the study. Furthermore, the sample was not random, collected by the snowball method over a limited period to quickly get an idea of the knowledge, attitude, and practice regarding COVID-19, in the current pandemic. Also, it is difficult to generalize our results to all Lebanese community pharmacists, since our sample consisted of more females and young pharmacists, comfortable in English, with easy access to social media: a selection bias is thus suspected, and our results may represent the best-case scenario of pharmacists’ information and practices. Pharmacists who did not participate might have poor access to social media and preventive information, particularly those intended for healthcare professionals. Although the questionnaire was piloted and adjusted before adopting the final version, it was developed on Google forms and made available online, thus restricting the participation of older pharmacists and those with little computer literacy and poor internet access. Nevertheless, the online questionnaire led to wide accessibility among pharmacists all over Lebanon; the anonymity of the questionnaire would also lead to a lower information bias, although a better measurement would be expected with validated scales. Based on the above, future studies conducted on a larger sample and using validated measurement scales would help the competent authorities design appropriately tailored interventions at the national level.

The strengths of this study include its originality and the fast response to identify KAP among community pharmacists in Lebanon, a Middle Eastern developing country where little information is available during this critical period. This study also complements other published works on KAP and preparedness of hospital pharmacists and nurses towards COVID-19 [28, 29]. To our knowledge, no studies were published on the practice of community pharmacists towards COVID-19 worldwide, particularly in Lebanon. Only one review narrated their activities in China, such as establishing remote pharmacy services to prevent human-to-human infections, providing event-driven pharmaceutical care, educating the public on infection prevention and disease management, and participating in clinical trials and drug evaluation [30].

## Conclusion

Our findings revealed an adequate level of knowledge and good practice towards COVID-19 among Lebanese community pharmacists. Their fears of contracting the virus and compromising the safety of those around them are justified. However, their supply of protective equipment is limited. The MOPH, professional organizations, and academic institutions in Lebanon should respond promptly to offer seminars/webinars and prepare brochures about COVID-19, as requested by community pharmacists. This study shows the need for the active involvement of relevant authorities to better prepare and involve pharmacists in situations of emergency.

## Supplementary Information

**Additional file 1.** Appendix A: Questionnaire.

## Data Availability

There is no public access to all data generated or analyzed during this study to preserve the privacy of participants.  The dataset that supports the conclusions is available upon request to the corresponding author..

## Abbreviations

COVID-19: The 2019 Coronavirus Disease
WHO: World Health Organization
MOPH: Ministry of Public Health
OPL: Order of Pharmacists of Lebanon
FIP: International Pharmaceutical Federation
