# Supplementary material for: A cross-sectional survey on community pharmacists readiness to fight COVID-19 in a developing country: knowledge, attitude, and practice in Lebanon
Source: J Pharm Policy Pract. 2021 Jun 11;14:51. doi: 10.1186/s40545-021-00327-6 (PMC8193173; doi:10.1186/s40545-021-00327-6)
Supplement: Supplementary file 1 — Additional file 1. Appendix A: Questionnaire. [file 40545_2021_327_MOESM1_ESM.docx]

**Appendix A: Questionnaire**

1. Date of filling the questionnaire: _____________

**Demographics**

1. Age: _______ years
2. Sex: Male Female
3. Mohafaza: Beirut Mount Lebanon North South Bekaa
4. Experience in community pharmacy: _______years
5. Job Owner Employee
6. Status Part time Full Time
7. Pharmacy surface area: …….m2
8. **Per day, how many patients come into the pharmacy asking you questions about the COVID-19 virus?**
9. <10
10. 10-50
11. 51-100
12. >100
13. **How many pharmacists/technicians are on duty at any single point in time at the pharmacy?**
14. 1
15. 2
16. 3
17. >3 specify _______

**SECTION I: KNOWLEDGE**

1. **Which of the following is true about COVID-19? (check all that applies)**

- Person to person transmission can occur by droplets
- Transmission can be airborne
- Most common signs and symptoms include fever, diarrhea and dyspnea
- I do not know

1. **Effective methods to protect yourself from potentially infected patients? (check all that applies)**

- Wearing surgical mask during the shift
- Washing hands frequently with soap and water
- Rubbing hands with alcohol-based gel
- Wearing gloves and changing them frequently
- Maintain a physical separation of at least 1.5m
- Limit the amount of time interacting with an individual who has respiratory symptoms
- I do not know

1. **For how long should a person be isolated in case of COVID-19 infection suspicion (mild symptoms or contact with an infected persons)?**
2. 7 days
3. 10 days
4. 14 days
5. 20 days
6. >20 days
7. I do not know
8. **Can someone who has been quarantined for COVID-19 spread the illness to others?**
9. No, if the quarantine period is less than 14 days
10. No, if the quarantine period is 14 days or more
11. I do not know
12. **What are the steps to take to protect yourself? (check all that applies)**

- Wash your hands with soap and water for at least 10 seconds
- Wash your hands with soap and water for at least 20 seconds
- Avoid close contact; put distance between yourself and other people (1.5-2 meters)
- Wear a facemask and stay home if you have any respiratory symptom
- No need to clean and disinfect solid objects (tables, doorknobs, desks, phones, etc.)

1. **Can a person test negative and later test positive for COVID-19?**
2. No
3. Yes
4. I do not know
5. **If a suspected person tests negative but has no symptoms (check all that applies):**
6. It is definitely a true negative
7. It can be a false negative in the pre-symptomatic phase
8. I do not know how to interpret this test result, I refer to a specialist
9. **Is the person at risk if he/she goes to a funeral of someone who died of COVID-19?**
10. Yes, since he will meet the dead person close contacts
11. No known risk currently
12. I do not know
13. **When can Confirmed COVID-19 cases be released from quarantine?**
14. Following one negative PCR test after resolution of symptoms
15. Following two negative PCR tests 24 hours apart after resolution of symptoms
16. Following four negative PCRs on three consecutive days after resolution of symptoms
17. I do not know
18. **Should I avoid contact with pets or other animals if I am sick with COVID-19?**
19. No
20. Yes
21. I do not know
22. **Which of the below products are you recommending to patients to disinfect? (Check all that applies)**
23. Alcohol 60%
24. Alcohol 70%
25. Alcohol 95%
26. **What are the most common symptoms related to COVID-19?**
27. Fever, productive cough, rhinorrhea
28. Fever, dry cough, dyspnea
29. Fever, diarrhea, pharyngitis
30. None of the above
31. I do not know
32. **Indicate which of the following options can be used to treat COVID 19 to date?**

- Acetaminophen
- Non-steroidal anti-inflammatory drugs (NSAIDS)
- Corticosteroids
- Symptomatic respiratory relief (inhalers)
- Lopinavir/ritonavir (initially for HIV)
- Chloroquine/remdesivir in combination
- Tocilizumab (initially for rheumatoid arthritis)

1. **Intravenous high dose vitamin C has been recommended in the treatment of COVID 19?**
2. True
3. False
4. I don’t know
5. **What is the MOPH hotline number?**
6. 112
7. 125
8. 1214
9. 1515
10. **Do you have time to get information regarding covid-19 outbreak?**
11. No
12. Yes
13. **Do you have time to get information regarding Covid-19 outbreak?**

3-4hours/day 1-2hours/day <1hour/day Not at all

1. **Where do you get your information on COVID-19 from? (Check all that applies)**

- CDC website
- Ministry of Public Health website
- World Health Organization
- Infectious Disease Society of America
- Media website/Internet
- Facebook
- Friends/family members
- Television
- Other, specify: ……………………………

**SECTION II: ATTITUDE**

**Kindly answer using a scale of Never to Always**

| **Questions** | **Never** | **Rarely** | **Often** | **Always** |
| --- | --- | --- | --- | --- |
| 1. Are you afraid of getting infected with COVID-19 due to occupational exposure? |  |  |  |  |
| 1. Are you afraid your family members get infected because of your occupational exposure? |  |  |  |  |
| 1. Do you feel depressed/exhausted due to the current pandemic? |  |  |  |  |
| 1. Are stress feelings affecting your duties (counseling, education, assessment)… |  |  |  |  |
| 1. Are stress feelings affecting your relationship with your staff and family members? |  |  |  |  |
| 1. Does any of your staff declare wanting to leave work due to COVID-19 fear? |  |  |  |  |
| 1. Do you implement specific icebreaking or energizing actions in your pharmacy to mitigate your staff stress? |  |  |  |  |

**Section III: Practice**

**Pharmacy Practice (Safety of Pharmacy Staff)**

| **Questions** | **No** | **Yes** |
| --- | --- | --- |
| 1. Was any of your staff members infected by COVID-19? |  |  |
| 1. Did you put a sign outside to restrict patients inside the pharmacy to one only at a time? |  |  |
| 1. Did you put a sign directing the patient not to enter the pharmacy in case of symptoms or of exposure? |  |  |
| 1. If yes, is this sign being read by patients? |  |  |
| 1. Did you make hand gel (hydro-alcoholic) available for patients to use before approaching the counter? |  |  |
| 1. Did you install a Plexiglas/Glass protective shield? |  |  |
| 1. Do you restrict the number of patients’ entering to the pharmacy |  |  |
| 1. Do you deliver medications through a window while forbidding all patients/clients from entering? |  |  |
| 1. Did you create any mark-up barrier for patients to stay away at least 1.5 meter from you? |  |  |
| 1. Are you wearing a mask while performing your job at the pharmacy |  |  |
| 1. Are you required to wear a mask while performing your job at the pharmacy |  |  |
| 1. Are you wearing gloves while performing your job at the pharmacy |  |  |
| 1. Are you required to wear gloves while performing your job at the pharmacy |  |  |
| 1. Are you wearing goggles/glasses to protect your eyes while performing your job at the pharmacy |  |  |
| 1. Are you still working as a full team as before COVID-19? |  |  |
| 1. If you are alternating schedule, were you asked to use vacation days? |  |  |
| 1. Did you organize yourself in a way to serve your patients outside the community pharmacy? (delivery for example) |  |  |
| 1. Do you prefer patients to pay by credit/debit card? |  |  |
| 1. Are you using a basket to collect money from patients to avoid direct contact with them? |  |  |
| 1. Did the working hours decrease for staff to decrease exposure? |  |  |
| 1. Did the working hours decrease for pharmacy owner to decrease exposure? |  |  |
| 1. Did you make rotation for your staff to decrease exposure? |  |  |
| 1. For how long do you put your face mask before changing it? |  | |
| 1. For how long do you put your gloves before changing them? |  | |

**Kindly answer using a scale of Never to Always**

| **Questions** | **Never** | **Rarely** | **Often** | **Always** |
| --- | --- | --- | --- | --- |
| 1. Are you able to wash your hands during your work shift? |  |  |  |  |
| 1. Are you able to rub your hands with hydro-alcoholic gel during your work shift? |  |  |  |  |
| 1. Are you able to maintain social distancing of at least 1.5m from patients |  |  |  |  |
| 1. Are you able to maintain social distancing of at least 1.5m from work colleagues |  |  |  |  |
| 1. Are you able to avoid touching eyes, nose and mouth? |  |  |  |  |
| 1. Are you able to avoid close contact with people/patients with respiratory symptoms? |  |  |  |  |
| 1. Are you able to stay home if you are not feeling well? |  |  |  |  |
| 1. Do you put gloves during your work shift? |  |  |  |  |
| 1. Do you put a mask during your work shift? |  |  |  |  |

**Pharmacy Practice (Service to patients)**

| **If a patient comes to your pharmacy with respiratory symptoms:** | **Never** | **Rarely** | **Often** | **Always** |
| --- | --- | --- | --- | --- |
| 1. Do you ask him questions about his travel history? |  |  |  |  |
| Do you ask the patient if he had contact with a confirmed case COVID-19 |  |  |  |  |
| Do you ask him if he had a contact with travelers coming from affected area |  |  |  |  |

**Pharmacy Practice (Service to patients)**

| **Questions** | **No** | **Yes** |
| --- | --- | --- |
| 1. Do you still have enough masks to sell to patients? |  |  |
| 1. If yes, are you selling them one by one/in small quantities? |  |  |
| 1. Do you still have enough gloves to sell to patients? |  |  |
| 1. If yes, are you selling them one by one/ in small quantities? |  |  |
| 1. Do you still have enough sanitizers to sell? |  |  |
| 1. If yes, are you selling them one by one/ in small quantities? |  |  |
| 1. Do you offer masks free of charge to patients? |  |  |
| 1. Are you facing any delay in the supply of masks, gloves or hand gels from the suppliers? |  |  |
| 1. Are you facing an increase in the price of the masks, gloves and hand gels from the supplier in a regular manner? |  |  |
| 1. Are you facing any pressure from the suppliers to pay on cash or in a short period of time? |  |  |
| 1. Do you offer awareness brochure to the patients? |  |  |

1. **What is the supplement most requested by patients? (Please check all that applies)**

- Vitamin C
- Vitamin D
- Multivitamin
- Supplement containing Zinc
- Other_____

**How do you behave in these situations? (Check all that applies)**

| **Situation** | **Call the**  **Physician** | **Call the**  **MOPH** | **Refer to**  **Laboratory Testing** | **Give preventive advice** |
| --- | --- | --- | --- | --- |
| 1. Flu-like symptoms with, during the last 14 days, history of travel to infected areas/ Or contact with a COVID_19 case |  |  |  |  |
| 1. Flu-like symptoms without history of travel to infected areas or contact with confirmed COVID-19 case |  |  |  |  |
| 1. Corona suggestive symptoms (fever, dry cough, dyspnea) |  |  |  |  |
| 1. Patient with a Positive COVID-19 PCR test, with or without respiratory symptoms |  |  |  |  |
| 1. Patient panicking due to a suspected exposure |  |  |  |  |
| 1. Patient panicking without symptoms or exposure |  |  |  |  |

**MOPH Hotline**

| **Questions** | **No** | **Yes** | **N/A** |
| --- | --- | --- | --- |
| 1. Did you try to reach for the MOPH Coronavirus call center |  |  |  |
| 1. Were you able to contact the MOPH call center/hotline when you need it? |  |  |  |
| 1. Did you contact any other institution for supportive information on COVID-19? |  |  |  |
| 1. Did you attend any awareness session on COVID-19? |  |  |  |

1. **If you called the MOPH hotline/call center, how useful was the information provided:**

Very useful Somehow useful Not useful Not applicable

1. **If you called other institutions for supportive information, how useful was the information provided:**

Very useful Somehow useful Not useful Not applicable

**Any other comment you would like to share**

_____________________________________________
